# Induction of apoptosis and inhibition of cell growth by *tbx5 *knockdown contribute to dysmorphogenesis in Zebrafish embryos

**DOI:** 10.1186/1423-0127-18-73

**Published:** 2011-10-08

**Authors:** Jenher Lu, Tzuchun Tsai, Sielin Choo, Shuyu Yeh, Renbing Tang, Anhang Yang, Hsinyu Lee, Jennkan Lu

**Affiliations:** 1 Department of Pediatrics and Pathology, Taipei Veterans General Hospital, Taipei, Taiwan; 2 School of Medicine, National Yang Ming University, Taipei, Taiwan; 3 Department of Medical Research and Education, National Yang-Ming University Hospital, Yilan, Taiwan; 4 Laboratory of Molecular Biology, Institute of Aquaculture, National Taiwan Ocean University, Keelung, Taiwan; 5 Institute of Zoology, National Taiwan University, Taipei, Taiwan

**Keywords:** zebrafish, mitochondria, apoptosis, *tbx5*, Holt-Oram syndrome, cell cycle

## Abstract

**Background:**

The tbx5 mutation in human causes Holt-Oram syndrome, an autosomal dominant condition characterized by a familial history of congenital heart defects and preaxial radial upper-limb defects. We report aberrant apoptosis and dormant cell growth over head, heart, trunk, fin, and tail of zebrafish embryos with tbx5 deficiency correspond to the dysmorphogenesis of tbx5 morphants.

**Methods:**

Wild-type zebrafish embryos at the 1-cell stage were injected with 4.3 nl of 19.4 ng of tbx5 morpholino or mismatch-tbx5-MO respectively in tbx5 morphants and mismatched control group. Semi-quantitative RT-PCR was used to for expression analysis of apoptosis and cell cycle-related genes. TUNEL and immunohistochemical assay showed the apoptosis spots within the local tissues. Ultra-structure of cardiac myocardium was examined by transmission electron microscope.

**Results:**

Apoptosis-related genes (bad, bax, and bcl2), and cell cycle-related genes (cdk2, pcna, p27, and p57) showed remarkable increases in transcriptional level by RT-PCR. Using a TUNEL and immnuohistochemical assay, apoptosis was observed in the organs including the head, heart, pectoral fins, trunk, and tail of tbx5 knockdown embryos. Under transmission electron microscopic examination, mitochondria in cardiomyocytes became swollen and the myocardium was largely disorganized with a disarrayed appearance, compatible with reduced enhancement of myosin in the cardiac wall. The ATP level was reduced, and the ADP/ATP ratio as an apoptotic index significantly increased in the tbx5 deficient embryos.

**Conclusion:**

Our study highlighted that tbx5 deficiency evoked apoptosis, distributed on multiple organs corresponding to dysmorphogenesis with the shortage of promising maturation, in tbx5 knockdown zebrafish embryos. We hypothesized that mesenchymal cell apoptosis associated with altered TBX5 level may subsequently interfered with organogenesis and contributed to dysmorphogenesis in tbx5 deficiency zebrafish embryos.

## 1. Background

*Tbx5 *belongs to the T-box family of transcription factors and is required for the embryonic development of the heart and forelimbs [1,2]. *Tbx5 *mutations in humans cause Holt-Oram syndrome (HOS), an autosomal dominant condition characterized by a familial history of congenital heart defects and preaxial radial ray upper-limb defects [3,4]. The phenotypic manifestations of *tbx5 *deficiency in different vertebrates are quite similar. *Tbx5 *deficiency in zebrafish causes multiple organ defects during organogenesis, including a shortened trunk, failure of cardiac looping formation, and hypogenesis or agenesis of the pectoral fins [5,6]. Either the type of mutation or the location of a mutation is predictive for the severity of heart or limb malformations in Holt-Oram syndrome patients. That is, there is no correlation between the *tbx5 *genotype and phenotype.

Since *tbx5 *encodes a transcription factor, *tbx5 *deficiency is presumed to disrupt development by altering the transcriptional function of the TBX5 protein, thereby affecting downstream target gene expressions, particularly affiliation binding proteins or transcription factors [7-9]. Though TBX5 is an important developmental regulator in normal development and disease, which TBX5 functions or whether the TBX5 protein plays a role as trasnscriptional activator or repressor, even divergent in different condition, is still relatively unclear.

The syndrome caused by mutations in T-box transcription factor 5 (TBX5) was described as "premature stops" or "developmental delay" in embryogenesis, though the mechanism how to delay or stop the development of the embryo, especially the cardiogenesis, remained indistinct [10]. Some revealed that the depletion of *tbx5 *leading to cardiac cell cycle arrest have been proved to elicit defect in cardiac sacromere formation and decreased cardiac cell number [11].

Apoptosis is a key event in many biological processes including embryogenesis and occurs in a variety of circumstance in specific time window in embryos. Zebrafish undergoes different levels of developmental cell death throughout embryonic stages precisely, including brain, spinal cord, ear, eyes, germ cells, tail bud and excretory system [12]. Apoptosis partakes in the complicate orchestration cell-cell interaction during organogenesis, such as cell differentiation in heart myocardium and the endocardial cushions during specific time [13]. Some postulated that anomalous apoptosis due to insufficient or improper induction of organogenesis specific factors leads to particular congenital diseases [14].

In this study, we reported that aberrant apoptosis and cell cycle arrest are increasing in zebrafish embryos with *tbx5 *deficiency; meanwhile, the excessive apoptosis is in coincidence with the dysmorphogenesis in individuals with *tbx5 *deficiency.

## Materials and methods

The approval of this study was granted by the National Taiwan Ocean University Aquaculture Animal ethics review board with annual evaluation.

### Maintenance of zebrafish

Zebrafish were maintained in 45-liter aquaria heated to 28.5°C with 25 fish per tank. The water was filtered, and about half of the water was replaced at least once a week. Adult zebrafish were fed 1 or 2 times per day with a variety of food, and the tank was cleaned by siphoning off any excess food after the second daily feeding. The day-night cycle was controlled with an automatic timer (14 h light/10 h dark).

### Breeding of zebrafish

Zebrafish reach sexual maturity in 10~12 weeks, but breeding fish should be between 7 and 18 months of age for maximum embryo production. The day before breeding, 1/3 of the water was replaced and the tank was cleaned after feeding (1~2 h before the end of the light period). Finally, a collection box was placed at the bottom of the tank and preparation was made to collect the embryos the next day.

### Embryo collection

When the light came on, we removed the collection box and placed the collected embryos into an incubator maintained at a temperature of 28.5°C.

### RNA isolation

Total RNA was isolated from 50 embryos using the guanidine isothiocyanate-based TRIzol solution. RNA samples were resuspended in DEPC-treated water and quantified spectrophotometrically at 260 nm. The RNA quality was then checked by 1.2% agarose gel electrophoresis, after staining with 1 μg/ml ethidium bromide. The RNA stock was stored at -80°C.

### Semi-quantitative Reverse-Transcriptase Polymerase Chain Reaction

Total RNA was prepared from 50 morpholino (MO) injected or normal embryos (Invitrogen Corporation, Carlsbad, CA, USA). Three microliters of 1st-strand cDNA was amplified. Amplification primers for each specific mRNA were deduced from published sequences and included *bcl2 *(P1: 5'-GTTCCACCCGTTTTCA-3', P2: 5'-GCGAGTCCTCATTCTGT-3'), *bad *(P1: 5'-CAAGCCTGGATAAACAC-3', P2: 5'-GGCAGATTGAAAGAAAG-3'), and *bax *(P1: 5'-AAGCATTGAGAGGTG-3', P2: 5'-AGAGGAAGTGAGGAGAA-3'), *p27 *(P1: 5'-GTTCGCTTGTCTAATGG-3', P2: 5'-GTCGGACTCAATGGTT-3'), *p57 *(P1: 5'-AGATTACGAGTGGGAGG-3', P2: 5'-TGAGTTCAGAGAGAAAGGG-3'), and *pcna *(P1: 5'-GCTCGCGGGATTTCT-3', P2: 5'-CAGCGGAGTGGCTTTGG-3'), *cdk2 *(P1: 5'-CAAGAGTTTCAGTCGC-3', P2: 5'-TAAGTCCGCACAGGTA -3'). PCR conditions were as follows: denaturation at 95°C for 3 min, followed by 50 cycles of amplification (95°C for 20 s, 59°C for 15 s, and 72°C for 20 s). Quantification of PCR product was performed on an electrophoresis agarose gel using Kodak image 3.5 software. All measurements were performed in triplicate (*n *= 3).

### Microinjection and morpholino treatment

The morpholino antisense oligonucleotide, *tbx5*-MO (5 GAAAGGTGTCTTCACTGTCCGCCAT-3), was designed against the *tbx5 *translational start site (Gene Tools LLC, Philomath, OR, USA) and a mismatch *tbx5*-MO (5'-GTCTCTTGACTCTCCGCGATCTCGG-3') was designed for control (Gene Tools LLC, Philomath, OR, USA). Wild-type embryos primarily at the 1-cell stage with the chorion intact were injected with 19.4 ng/4.3 nl of stock morpholino diluted in Danieau's solution. The injected embryos were raised at 28.5°C. Embryos used for analyzing the expression of various markers were fixed with 4% paraformaldehyde. Otherwise, embryos were scored after 2 days of development for late effects. In our previous study, three control groups, including 3' end of *tbx5*-MO(2) (5'-GCCTGTACGATGTCTACCGTGAGGC-3') and embryos with blank microinjection and wild-type ones without microinjection, were included to identify the specific blockage of *tbx5 *mRNA translation effect of *tbx5*-MO.^6 ^In order to examine the knockdown effectiveness of the *tbx5*-MO, a *tbx5*-GFP construct was generated by fusing a 468-bp fragment of *tbx5*, which included the morpholino target side into a GFP producing vector. *Tbx5*-MO at 19.4 ng and *tbx5*-GFP DNA at 150 μg were co-injected into 1-cell stage embryos. In control group, only 150 μg of *tbx5*-GFP was microinjected into 1-cell stage embryos [6].

### Immunohistochemical analysis

Zebrafish embryos were fixed with 4% paraformaldehyde in phosphate-buffered saline (PBS). Deparaffinized sections (3 μm) of zebrafish embryo tissues were placed on slides and processed for immunohistochemistry. After blocking with a biotin blocking system (Dako A/S, Glostrup, Denmark) for 30 min, sections were incubated with target-purified rabbit primary antibodies, including BCL2 (Anaspec Inc., Fremont, CA, USA), BAD (Anaspec Inc., Fremont, CA, USA), CASPASE-3 (Anaspec Inc., Fremont, CA, USA), CASPASE-8 (Anaspec Inc., Fremont, CA, USA),

CDK2 (Anaspec Inc., Fremont, CA, USA), P27 (Anaspec Inc., Fremont, CA, USA) and MF20 (Developmental Studies Hybridoma Bank, Iowa City, IA, USA), washed in PBS, and then incubated with a rhodamine-conjugated secondary antibody: goat anti-rabbit immunoglobulin G (IgG). After washing in PBS, sections were incubated with mounting medium and kept at 4°C.

### Transmission electron microscopic examinations

Embryos were fixed at 48 h post-fertilization (hpf) with 2.5% glutaraldehyde in Sorenson's phosphate buffer, postfixed with 1% OsO_4 _in Sorenson's phosphate buffer followed by dehydration through a graded series of ethanol washes, and embedded in Spurr's EPON. Blocks were heated in an oven for 8 h at 70°C. Semithin (1-μm) sections were cut and stained with toluidine blue for adequate preview under a microscope. Ultrathin sections (900 Å) were cut with a diamond knife, stained with uranyl acetate and lead citrate, and examined with an electron microscope.

### TUNEL assay

Both whole mount and sectioned TdT-UTP nick end labeling (TUNEL) assays were performed using an ApopTag kit (Chemicon, Heule, Belgium). Briefly, zebrafish embryos were fixed with 4% paraformaldehyde in PBS. Proteinase K-treated whole embryos or deparaffinized sections (5 μm) of embryos were incubated with the TdT enzyme followed by anti-digoxigenin. Finally, embryos or slides were stained with DAB for 5 min.

### Western blot analysis

Embryos were homogenized on ice in lysis buffer (Sigma-Aldrich, St. Louis, MO, USA). Then, cellular debris was pelleted by centrifugation at 12,000 rpm for 20 min, and the supernatant was collected and measured. Proteins were mixed with sample buffer before separating in 10% sodium dodecylsulfate polyacrylamide gel electrophoresis (SDS-PAGE) gels. The SDS-PAGE was then transferred onto nitrocellulose membranes at 100 V for 1 h. Membranes were blocked with blocking buffer (5% bovine serum albumin (BSA)) at room temperature for 1 h. The TBX5 primary antibody (Aviva Systems Biology LLC, San Diego, CA, USA) was incubated overnight at 4°C using the appropriate dilution (1: 1000). The nitrocellulose membranes were washed with PBST followed by incubating with a horseradish peroxidase (HRP)-conjugated secondary antibody (1: 5000) for 1 h at room temperature before the images were digitized.

### ADP/ATP Ratio

ATP and ADP/ATP ratio assay were performed using an ApoSENSOR™ ADP/ATP Ratio Assay Kit (Biovision, Mountain View, CA, USA). Ten embryos (*n *= 10 in each subgroup) were separately placed in 96-well round-bottomed, flat, white plates for detection. The RLU (relative luminescence unit) value was detected using the PARADIGM™ Detection Platform (Beckman Coulter Inc., Brea, CA, USA).

### Statistical analysis

Results are given as the mean ± S.D. Where applicable, a *t*-test was performed. Statistical significance was accepted at *p *< 0.05.

## Results

### Morphological characteristics of *tbx5 *knockdown embryos

**Figure 1:**
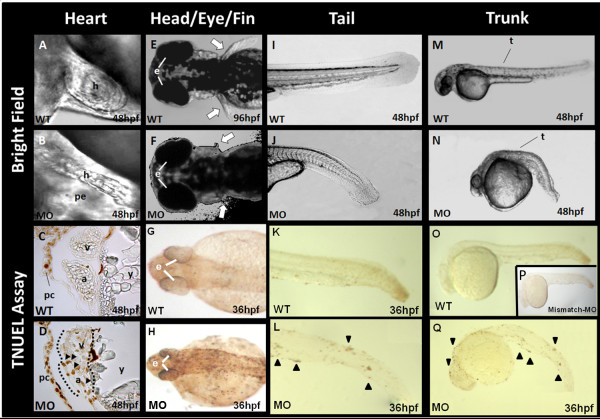
**Morphological changes and the TUNEL assay of *tbx5 *knockdown embryos**. A: In wild-type (WT) embryos, the atrium and ventricle overlap due to normal looping formation. The heart is located in the cardiac sac. B: In *tbx5 *knockdown embryos, a string-like cardiac morphology was observed, with no looping formation. Most of the cardiac sac was occupied by pericardial effusion. C: No apoptosis was observed in the atria or ventricle of WT embryos, but a little apoptosis was noted in the pericardium. D: Apoptosis occurred in the heart of *tbx5 *knockdown embryos accompanied by significant apoptosis in the pericardium. E-F: Symmetrical development of bilateral pectoral fins was observed in WT embryos (E); however, pectoral fins of *tbx5 *deficient embryos were hypoplasia (F). G: Trivial apoptosis was observed in the eyes, head, and the bilateral paravertebral mesenchymal region of WT embryos. H-J: Prominent apoptosis was detected in knockdown embryos (H). Compared to WT embryos (I, M), a shortened and curled malformed trunk was present in *tbx5 *knockdown embryos (J, N). K-Q: Prominent apoptosis was located along the curled tail (L) and trunk (Q) of *tbx5 *deficiency embryos, while normal embryos and mismatch-*tbx5*-MO injected embryos had a straight tail (K) and trunk (O, P). A-Q: The anterior of the embryos is to the left. A-B, I-Q: Lateral view; E-F: dorsal view; G-H: anterior view; C, D: Sagittal section. h, heart; pe, pericardial effusion; a, atrium; v, ventricle; y, yolk; t, trunk; white arrow, pectoral fin; black triangle, location of apoptosis; hpf, hour post fertilization; MO, *tbx5*-MO treated embryos.

The normal cardiac looping process was completed in wild-type (WT) embryos at 48 hpf, meanwhile the ventricle overlapped with the atria during the diastolic phase (Figure 1A). Injection of the *tbx5 *morpholino (MO) directly into embryos at the 1-cell stage resulted in retardation of the looping process, and the most prominent cardiac finding in *tbx5 *morphants was a string-like heart (Figure 1B). At 96 hpf, WT embryos had a pair of well-formed pectoral fins (Figure 1E); however, the pectoral fins of *tbx5 *knockdown embryos showed hypoplasia or even agenesis (Figure 1F). The tail (Figure 1J) and trunk (Figure 1N) of knockdown embryos were shortened and distorted in shape, while WT embryos had a straight tail (Figure 1I) and trunk (Figure 1M), both of which were longer.

### TUNEL assay of *tbx5 *knockdown embryos

We examined apoptosis using a TUNEL assay. Aberrant poptosis was present in the entire paravertebral mesenchymal zone of *tbx5 *knockdown embryos (Figure 1H, Q). Apoptosis was noted in the heart (Figure 1D), tail (Figure 1L), bilateral paravertebral mesenchymal region (Figure 1H) and trunk (Figure 1Q) of *tbx5 *knockdown embryos. Apoptosis also slightly noted in the pericardium (Figure 1C), tail (Figure 1K), bilateral paravertebral mesenchymal region (Figure 1G) and trunk (Figure 1O) of WT embryos. Furthermore, there was no significant difference between WT and mismatch-*tbx5*-MO treated embryos (Figure 1P).

### Molecular characteristics of *tbx5 *knockdown embryos

**Figure 2:**
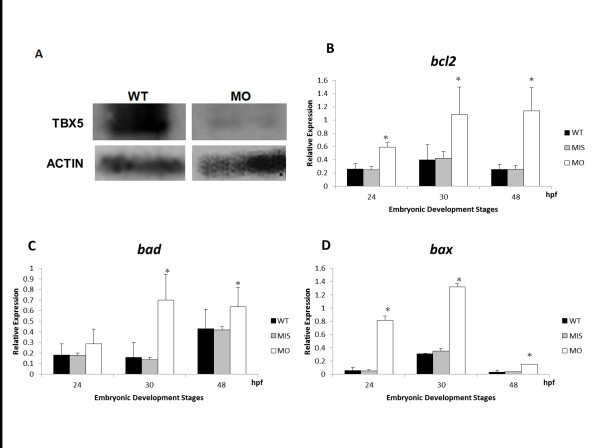
**Quantification of apoptosis-related gene expressions in *tbx5 *deficiency zebrafish embryos using a semi-quantitative PCR**. A: The efficiency of the *tbx5*-MO was tested using Western blot. *Tbx5*-MO remarkably inhibited the expression of the TBX5 protein (*n *= 3, 100 embryos). B-C: The proapoptotic genes, *bad *(B) and *bax *(C), were significantly activated in *tbx5*-MO-treated embryos. D: The antiapoptosis gene, *bcl2*, was significantly induced in *tbx5 *knockdown embryos throughout the developmental stages (*n *= 3, 50 embryos/stage; relative expression, gene expression/β-actin expression). Data are presented as the mean ± S.D. An asterisk indicates a significant difference (*p *< 0.05).

*Tbx5 *knockdown zebrafish embryos were used by microinjecting 19.4 ng/4.3 nl of *tbx5*-MO at 0 hpf as described in our previous study [6]. The efficiency of the *tbx5*-MO was confirmed both with Western blotting (Figure 2A) and *tbx5*-green fluorescent protein (GFP) construct [6].

We tested the expressions of apoptosis-related genes at 3 important developmental periods in both WT and MO-treated zebrafish embryos: heart tube formation at 24 hpf, looping formation at 30 hpf, and chamber formation at 48 hpf. Cell apoptosis-related genes, including *bad*, *bax*, and *bcl2*, showed remarkable increases in *tbx5 *morphants in all studied periods but not in embryos injected with mismatch-*tbx5*-MO, which has no significant different with the wild-type and control mismatch (Figure 2B-D).

**Figure 3:**
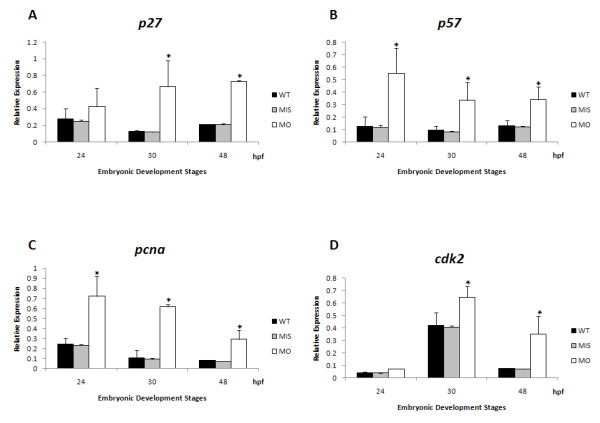
**Quantification of cell cycle-related gene expressions in *tbx5 *deficiency zebrafish embryos using a semi-quantitative PCR**. A-D: The cell cycle regulatory genes *p27 *(A), *p57 *(B), *bad *(C) and *bax *(D), were significantly activated in *tbx5*-MO-treated embryos. (*n *= 3, 50 embryos/stage; relative expression, gene expression/*b-actin *expression). Data are presented as the mean ± S.D. An asterisk indicates a significant difference (*p *< 0.05).

By semi-quantitative PCR methods, the depletion of *tbx5 *caused an increase of the expression of S stage-related mRNA: *p27 *and *p57 *(Figure 3A, B), and the significant increases in *cdk2 *and *pcna *(Figure 3C, D) expressions in *tbx5 *morphants. It suggested prolongation of the G1/S phase, which imply interfered cell cycle and reduction in cell number in *tbx5 *morphants.

### Immunohistochemical analysis

**Figure 4:**
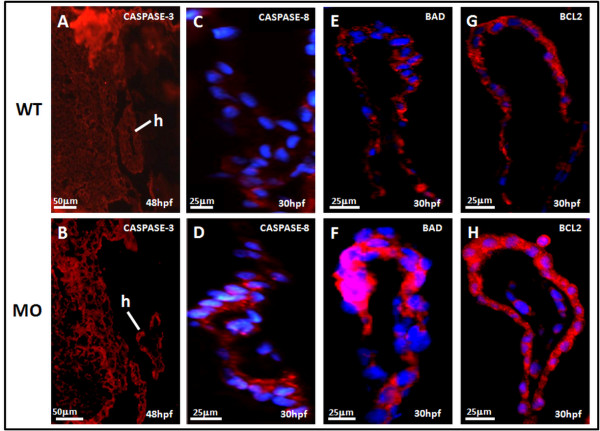
**Sagittal view of the heart in *tbx5 *knockdown embryos by immunohistochemical studies of heart**. A-B: Caspase-3 (red) expression could been slightly seen in wild-type (WT) embryos (A), but caspase-3 was more induced in knockdown embryos at 48 h post-fertilization (hpf) though the expression was not so strong (B). Caspase-8 (blue) was more induced in knockdown embryos (D) at 48 h post-fertilization (hpf) than the expression in wild-type (C). The proapoptotic factor, BAD (red), was enhanced in *tbx5 *deficiency embryos (F) with the nucleus stained by DAPI (blue); but there was almost no BAD expression in WT embryos (E). Compared to the WT (G), embryos with *tbx5 *deficiency showed a significant increase in BLC2 expression (H). The anterior of embryos is to the left. h, heart; MO, *tbx5*-MO-treated embryos.

The apoptosis downstream factor, CASPASE-8 and CASPASE-3, plays vital role in both cell intrinsic and extrinsic apoptosis. CASPASE-8 and CASPASE-3 was induced in the heart of *tbx5 *knockdown embryos at 48 hpf, which suggests the occurrence of apoptosis which might via activation of CASPASE-8 and therefore activates its downstream factor CASPASE-3 (Figure 4A-D).

**Figure 5:**
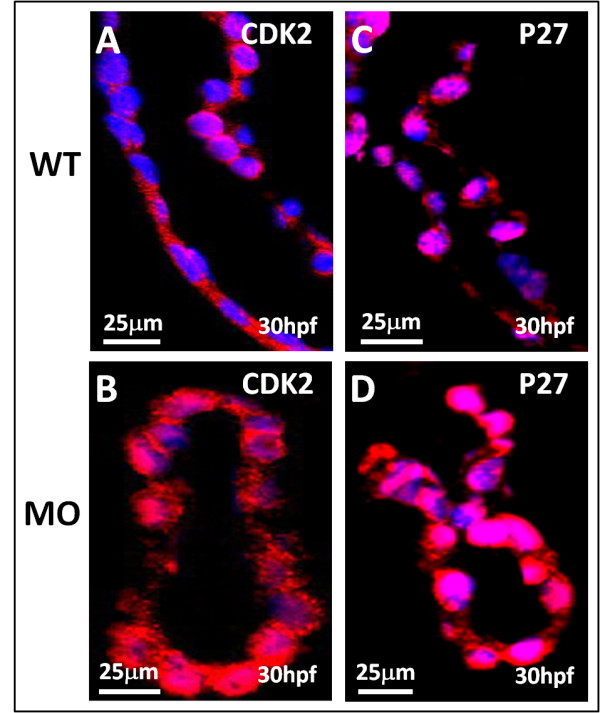
**Immunohistochemical studies of heart sagittal sections in *tbx5 *knockdown embryos by cell cycle-related antibodies**. A-D: Both cell cycle-related factor CDK2 (red) (A) and P27 (red) (C) expressed mildly in WT embryos but was highly induced in *tbx5 *knockdown embryos (B, D) at 30 hpf. The nucleus was counterstained with DAPI. The anterior of embryos is to the left. h, heart; MO, *tbx5*-MO-treated embryos.

**Figure 6:**
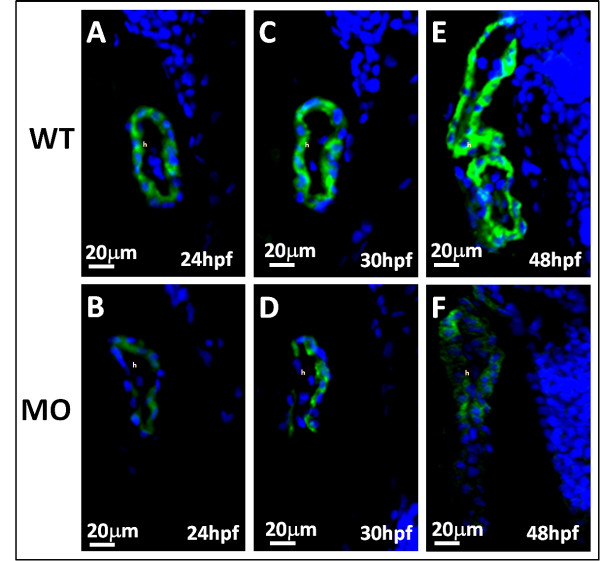
**Myosin of zebrafish embryos was stained by the heart-specific anti-myosin antibody, MF20 (green), and counterstained by DAPI (blue) for nuclear observation**. A-F: The expression of MF20 in embryos with *tbx5 *deficiency (*B*, *D*, *F*) decreased throughout the developmental stages compared to that in the WT (*A*, *C*, *E*), from 24 to 48 hpf. The anterior of embryos is to the left. h, heart; MO, *tbx5*-MO-treated embryos.

The overexpression of the apoptosis-related and cell-cycle-related protein, BAD (Figure 4E, F), BCL2 (Figure 4G, H), CDK2 (Figure 5A, B), P27 (Figure 5C, D) was confirmed by an immunohistochemical analysis. Cardiomyogenesis plays vital roles in different stages includes heart looping, chamber formation, and ballooning. Our study demonstrated that the expression of myosin, stained by the anti-cardiac myosin antibody, MF20, was reduced in *tbx5 *knockdown embryos (Figure 6A-F).

### Ultrastructural changes in mitochondria

**Figure 7:**
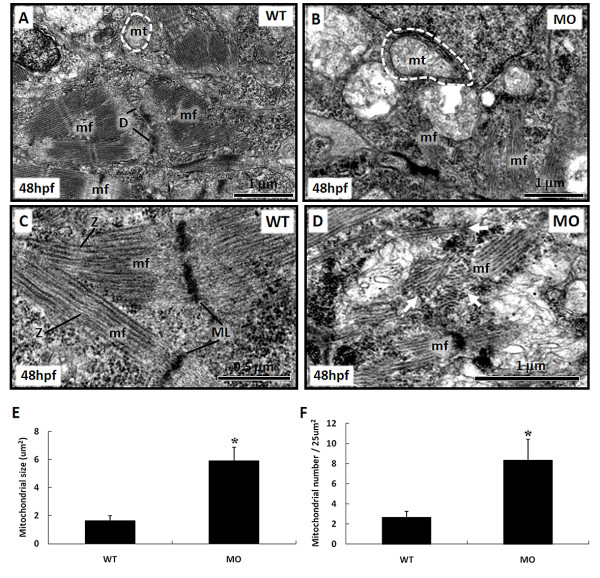
**Ultrastructural changes of mitochondria and myofibrils in the heart of *tbx5*-knockdown and wild-type (WT) embryos at 48 h post-fertilization (hpf) under TEM**. A: Small mitochondria and well-formed desmosomes can be observed in WT embryos. B: Multiple swollen mitochondria can be observed within myocytes of *tbx5 *knockdown embryos. C: Well-organized myofibrils with a M line and Z disc can be observed in normal embryos. D: Disarrayed myofibrils appeared in *tbx5 *knockdown embryos while the M line and Z disc were unrecognizable. E-F: Both the size (E) and number (F) of mitochondria had significantly increased in *tbx5 *knockdown embryos. Data are presented as the mean ± S.D. An asterisk indicates a significant difference (*p *< 0.05). mf, myofibril; mt, mitochondria; D, desmosome; ML, M-line; Z, Z-disc; white arrow, disarrayed myofibrils; MO, *tbx5 *knockdown embryos.

The mitochondrion is the center of the cell intrinsic apoptosis pathway, and the ultrastructure of mitochondria was markedly changed in *tbx5 *morphants. Mitochondria are normally compact in WT group (Figure 7A) but become swollen in *tbx5 *knockdown embryos (Figure 7B). The mitochondrial size of WT embryos was 1.64 ± 0.4 μm^2^, while swollen mitochondria were 5.91 ± 1 μm^2 ^(Figure 7E). Additionally, the amount of mitochondria remarkably increased in *tbx5 *knockdown embryos (8.33 ± 2.1 vs. 2.67 ± 0.6/25 μm^2^) (Figure 7F).

### Ultrastructural changes in cardiac myofibrils

Normal cardiac myofibrils were well organized as the Z-disc and M-lines in WT embryos at 48 hpf (Figure 7C). In contrast, the ultrastructure of the myocardium appeared largely disorganized in *tbx5 *knockdown embryos. In addition, myofibrils appeared to be disarrayed (Figure 7D). Furthermore, cardiocytes were disaggregated and failed to develop a tight junction to each other. (Figure 7A-B) The myofibril layer in the cardiac chamber wall had become very thin with a disarrayed arrangement (data not shown).

### Changes in the ADP/ATP ratio

**Figure 8:**
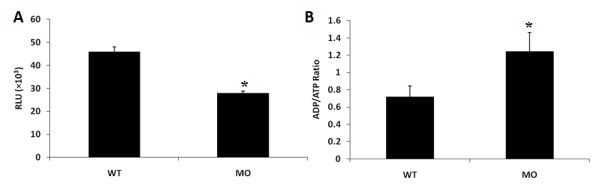
**Changes in the ADP/ATP ratio in *tbx5 *knockdown embryos**. A: In wild-type (WT) embryos, the ATP level was higher than that in *tbx5 *deficiency embryos. B: The ADP/ATP ratio of *tbx5 *knockdown embryos was significantly higher, indicated that apoptosis had occurred (*n *= 10). Data are presented as the mean ± S.D. An asterisk indicates a significant difference (*p *< 0.05). MO, *tbx5 *knockdown embryos.

The ratio of ADP/ATP has been used to distinguish if cells have undergone cell death or proliferation; an increase in the ADP/ATP ratio is recognized in an apoptotic state. We analyzed both the ATP level and ADP/ATP ratio in *tbx5 *deficient embryos at 48 hpf. The ATP level of *tbx5 *knockdown embryos (*n *= 10 in each subgroup) was remarkably decreased (Figure 8A). Furthermore, a high ADP/ATP level in *tbx5 *knockdown embryos indicated the occurrence of apoptosis (Figure 8B).

## Discussion

Organogenesis is a vital process during embryonic development and is often accompanied by apoptosis in certain time windows. Vertebrate embryos, including those of zebrafish, undergo programmed cell death throughout the development process [15,16]. Development of an organ or tissue is often preceded by the extensive proliferation and differentiation of a particular cell; the resultant mass is then "pruned" into the correct form by apoptosis. Therefore, embryonic development is strictly regulated by processes including cell proliferation, migration, differentiation, and apoptosis. Dysregulation of any of the processes can cause congenital diseases and anomalies. Previous evidence showed the important roles of apoptosis in embryonic cardiac development. Poelmann *et al*. reviewed how apoptosis occurs in the myocardium of the outflow tract and in the formation of the atrioventricular cushion, pulmonary trunk, semilunar valves, and even vascular remodeling [13]. Aberrant patterns, overactivation, or disruption of apoptosis can cause congenital malformations. For example, disorder of the apoptosis process impelled the regression of four-arch segments and interrupted aortic arch type B in tumor growth factor (TGF)-β2-deficient mice [17].

Our data revealed activation of apoptosis and inhibition of cell growth in embryonic *tbx5 *morphants at 36 hpf by transcriptional and translational levels. By RT-PCR, the members of BCL-2 family genes and cell cycle-related genes were overexpressed in specific time window during zebrafish embryonic development. All apoptosis related genes either anti-apoptosis related gene (*bcl-2*) or pro-apoptosis related genes (*bad *and *bax*) were all transcriptionally activated, and so were the cell proliferation markers (*cdk2 *and *pcna*) and cyclin kinase inhibitors (*p27 *and *p57*). In protein level, remarkably plentiful production of apoptosis-related proteins including CASPASE-3, CASPASE-8, and BAD were proved by immunohistochemistry These data disclose that a deficiency of *tbx5*, a transcription factor, in zebrafish embryos may trigger multiple signal pathways including promoting or inhibiting molecules and eventually come to the end sequel of apoptosis and cease of cell growth.

Besides, the distribution of aberrant apoptosis and deviant cell cycle caused by a deficiency of *tbx5 *in the embryos might be associated with the dysmorphogenesis, especially engendering the string-like heart, short fins, and malformed trunk during zebrafish embryonic development.

There are two main apoptosis pathways including cell-intrinsic (mitochondrial) pathway and cell-extrinsic (death receptor) pathway; the intrinsic pathway is largely regulated by mitochondria, whereas the extrinsic pathway interacts with death receptors [18]. Mitochondria play fundamental roles in both life and death to provide factories of bio-energy and also gates of apoptosis for cells [19]. There is no exception in embryonic development. Mitochondria supply energy to embryos from the beginning of fertilization to ensure that the progressive development of an embryo is clearly documented [20,21]. The synthesis of ATP which is compulsory for maintaining regular cellular function was impaired during the mitochondrial apoptotic process and diminished cell viability [22-24]. In addition, resynthesis of ATP decreased in the mitochondria of apoptotic cells, and a relatively low ratio of ATP to ADP can be used to monitor apoptosis. The decreased ATP level suggests an inadequate energy supplement; furthermore, conversion of the ADP/ATP ratio indicates energetic exhaustion as a result of an apoptotic process in *tbx5*-deficient embryos at the late stage of organogenesis [25,26].

The mitochondrial apoptotic process causes the outer membrane of mitochondria to release proapoptotic proteins and eventually gives rise to swollen mitochondria [27,28]. Our ultrastructural findings by TEM revealed that mitochondria were swollen and loose in the hearts of *tbx5 *knockdown embryos, rather than small and dense in WT embryos. This phenomenon is compatible with the morphological changes of mitochondria during apoptosis, which culminates in permeabilization of the mitochondrial outer membrane and release of soluble proteins from the mitochondrial intermembrane space. Once the mitochondrial permeability transition pore is opened, proapoptotic proteins are released, and the membrane potential collapses, which results in swelling of mitochondria. The number of mitochondria in *tbx5 *deficiency embryos also increased in addition to their size. Although a similar phenomenon was described during apoptosis induced by anticancer drugs in colon cancer cells and apoptosis in mitochondrial myopathy, mitochondrial proliferation accompanied by mitochondrial swelling was first reported in apoptosis of zebrafish embryos with *tbx5 *deficiency [29]. Until now, the role of mitochondrial proliferation linked to apoptosis has remained unexplained.

Under TEM, the ultrastructure of the myocardium in *tbx5 *knockdown embryos appeared eminently disorganized. The myofibrils were quantitatively diminished at both the atrial and ventricular levels. We used MF20, an anti-myosin antibody, to prove a decrease in cardiac myosin. This result is in accordance with conclusion of our previous study that cardiomyogenesis-related genes were perturbed in *tbx5 *knockdown embryos [6].

The TUNEL assay showed that a shortage of *tbx5 *activated aberrant apoptosis and dormant cell growth in the head, heart, fins, and trunk. Although abnormal apoptosis-related genes were expressed throughout the entire process of organogenesis in *tbx5 *knockdown embryos, the degree and distribution of apoptosis varied in different locations at 48 hpf. Such aberrant apoptosis may contribute to consequent morphologic anomalies including a string-like heart, a curled trunk, and hypogenesis of the pectoral fins, but it did not interfere with the migration and differentiation of progenitor cells during early development. For example, no ectopic fin was found in embryos, although hypogenesis or agenesis of the pectoral fin was frequently observed in *tbx5 *morphants, as was the string-like heart. We hypothesized that the aberrant apoptosis may play a role in the interruption of cardiac looping, chamber formation, and growth of pectoral fins in zebrafish. Nevertheless, mutations in *tbx5 *gene underlie Holt-Oram syndrome, the majority of which is assumed as sequel "premature stops" or "developmental delay", may induce inappropriate apoptotic processes during embryogenesis and lead to the dysmorphogenesis including cardiac and limb malformation.

From a genomic analysis, dozens of genes were related to the underlying causes of isolated congenital heart disease and genetic syndrome associated with cardiac anomalies. However, genotype-phenotype distinctions exist. The discrepancy was frequently related to multiple genetic pathways, the size of the deletion, different allele/loci, point mutations, or a haploinsufficiency. Our observation presumed that the genotype-phenotype distinction might be a consequence of variable degrees of apoptosis in progenitor cells.

Some study declared that exogenous TBX5 could induce apoptosis and inhibit cell proliferation in vitro [30]. Our results revealed *tbx5 *insufficiency ultimately resulted in activation of apoptosis and inhibition of cell growth in whole individual of zebrafish embryo, though knockdown of TBX5 leads to overexpression of both anti-apoptotic and pro-apoptotic gene than wild type embryos and the controls. Those data implied that abnormal *tbx5 *level play a role in aberrant apoptosis and cell growth contributing to dysmorphogenesis.

## Conclusion

In summary, abnormal appearances in zebrafish embryos with *tbx5 *deficiency including string-like heart, curled trunk, curled tail, and pectoral fins might be a consequence of aberrant apoptosis and dormant cell growth in early embryonic development, which affected late differentiation and growth of organs as well as maturation.

## Competing interests disclosure

The authors declare that they have no competing interests.

## Authors' contributions

JHL and JKL conceived of the study, participated in its design, coordination and drafted the manuscript. TT participated in its design and drafted the manuscript, SC, SY and AY carried out the molecular genetic studies. RT and HL participated in its design and coordination. All authors read and approved the final manuscrpt.
